# Arterial spin labeling MRI in patients undergoing carotid artery revascularization: a systematic review of the hemodynamic changes and clinical implications

**DOI:** 10.1007/s00330-025-11885-7

**Published:** 2025-08-06

**Authors:** Alessandro Carrozzi, Elia Manfrini, Carlo Golini, Luigi Vincenzo Pastore, Annalisa Vitale, Pietro Bartolo, Manuel Requena, Francesco Diana, Marta de Dios Lascuevas, Claudia Testa, Luigi Cirillo, David Hernández, Cristina Auger, Àlex Rovira, Alejandro Tomasello, Laura Ludovica Gramegna

**Affiliations:** 1 Department of Medical and Surgical Sciences (DIMEC), University of Bologna, Bologna, Italy; 2 Department of Radiology, University of Cagliari, Cagliari, Italy; 3 Department of Physics and Astronomy (DIFA), University of Bologna, Bologna, Italy; 4 UOC Neuroradiologia, “Umberto I” Hospital, Nocera Inferiore, Italy; 5 Neuroradiology Section, Vall D’Hebron University Hospital, Barcelona, Spain; 6 Interventional Neuroradiology Section, Vall D’Hebron University Hospital, Barcelona, Spain; 7 Vall d’Hebron Research Institute (VHIR), Vall d’Hebron Barcelona Hospital Campus, Barcelona, Spain; 8 Department of Scienze della Vita, Della Salute e delle Professioni Sanitarie, Link Campus University, Rome, Italy; 9 UOC Neuroradiologia, IRCCS Istituto delle Scienze Neurologiche di Bologna, Bologna, Italy; 10 Department of Biomedical and Neuromotor Sciences (DIBINEM), University of Bologna, Bologna, Italy; 11 Servicio de Radiología, Unidad de Neurorradiología, Hospital del Mar, Barcelona, Spain

**Keywords:** Carotid artery stenosis, Arterial spin labeling MRI, Cerebral blood flow, Carotid revascularization, Cerebral hyperperfusion syndrome

## Abstract

**Objectives:**

Arterial spin labeling (ASL) MRI is a non-invasive imaging modality that measures cerebral blood flow (CBF) without the need for contrast agents or radiation, offering insights into hemodynamic changes. Carotid revascularization procedures, carotid endarterectomy and carotid artery stenting, aim to improve cerebral perfusion and reduce the risk of ischemic events. This study explores ASL’s clinical potential in assessing CBF changes in carotid stenosis patients prior to revascularization procedures.

**Materials and methods:**

A systematic review was conducted following PRISMA guidelines to identify studies that utilized ASL in patients undergoing carotid revascularization. Searches were performed in the MEDLINE/PubMed and Web of Science databases. Extracted data included patient demographics, ASL acquisition parameters, perfusion analysis methods, and study findings related to ASL results.

**Results:**

Twenty studies involving 710 patients were included. Preoperative ASL consistently identified perfusion deficits ipsilateral to stenosis, which improved post-revascularization, particularly in eloquent brain regions. After revascularization, CBF increase was greatest in patients with severe baseline deficits and smaller in those with prior strokes. ASL metrics predicted post-procedural cerebral hyperperfusion (CH), though protocol variability influenced results. Visual assessment methods based on arterial transit artifacts (ATA) emerged as practical tools for hyperperfusion risk prediction without requiring extensive post-processing.

**Conclusion:**

ASL MRI is a valuable tool for assessing hemodynamic changes in carotid artery stenosis and predicting treatment outcomes, particularly the risk of hyperperfusion. Its non-invasive nature and ability to evaluate collateral flow enhance its clinical value.

**Key Points:**

***Question*** *Can arterial spin labeling (ASL) MRI reliably assess cerebral blood flow changes in patients with carotid stenosis undergoing revascularization, improving decision-making regarding risks and outcomes?*

***Findings*** *ASL detects pre-treatment perfusion deficits, quantifies post-revascularization blood flow increases, predicts hyperperfusion risk, and assesses collateral flow in carotid stenosis patients undergoing endarterectomy or stenting.*

***Clinical relevance*** *ASL MRI provides a non-invasive method to evaluate cerebral perfusion in carotid stenosis, aiding in risk assessment for cerebral hyperperfusion syndrome and optimizing treatment strategies by preoperatively assessing collateral circulation and post-treatment cerebral blood flow recovery.*

## Introduction

Arterial spin labeling (ASL) MRI is a non-invasive technique that measures brain perfusion without requiring intravascular contrast agents and ionizing radiation [1]. This method uses the body’s water molecules in the arteries as natural tracers, magnetically labeling them to track blood flow to specific organs [2], particularly the brain. In ASL, selective radiofrequency pulses invert the magnetization of water in the arterial blood, typically applied at the neck for brain imaging. Subsequent imaging captures the movement of these labeled water molecules as they enter and spread through brain tissue.

ASL assesses brain perfusion in terms of cerebral blood flow (CBF), defined as the volume of blood passing through a specific amount of brain tissue per unit of time, usually measured in milliliters per minute per 100 grams of tissue. ASL can be acquired using three primary techniques: pulsed ASL (PASL), continuous ASL (CASL), and pseudo-continuous ASL (pCASL), with the latter having emerged as the modality of choice for clinical applications due to its superior signal-to-noise ratio. Additionally, vessel encoding ASL—a specialized technique capable of assessing the vascular territory of individual vessels—has been developed [3, 4].

Extracranial carotid artery stenosis is a significant risk factor for stroke [5], and alterations in cerebral hemodynamics affecting brain perfusion are associated not only with an increased risk of stroke but also with chronic vascular damage and cognitive decline. Carotid revascularization treatments, including carotid endarterectomy (CEA) and carotid artery stenting (CAS), play a crucial role in managing extracranial carotid artery stenosis or occlusion by reducing the risk of ischemic strokes and improving overall cardiovascular health [6].

Several technical challenges remain in the clinical use of ASL for patients with carotid artery stenosis. First, uncertainties persist regarding the placement of the tagging plane perpendicular to the neck vessels. Another major challenge is selecting the optimal post-labeling delay (PLD), a critical parameter that defines the time between labeling arterial blood and image acquisition for CBF measurement. In patients with prolonged arterial transit times, such as those with carotid artery stenosis, an insufficient PLD can result in a significant underestimation of CBF. Conversely, a PLD that is too long leads to signal loss due to the T_1_ decay of labeled blood. Additionally, if the PLD is too short, artifacts may arise because the labeled blood remains within the arteries during image acquisition, compromising measurement accuracy.

**Fig. 1:**
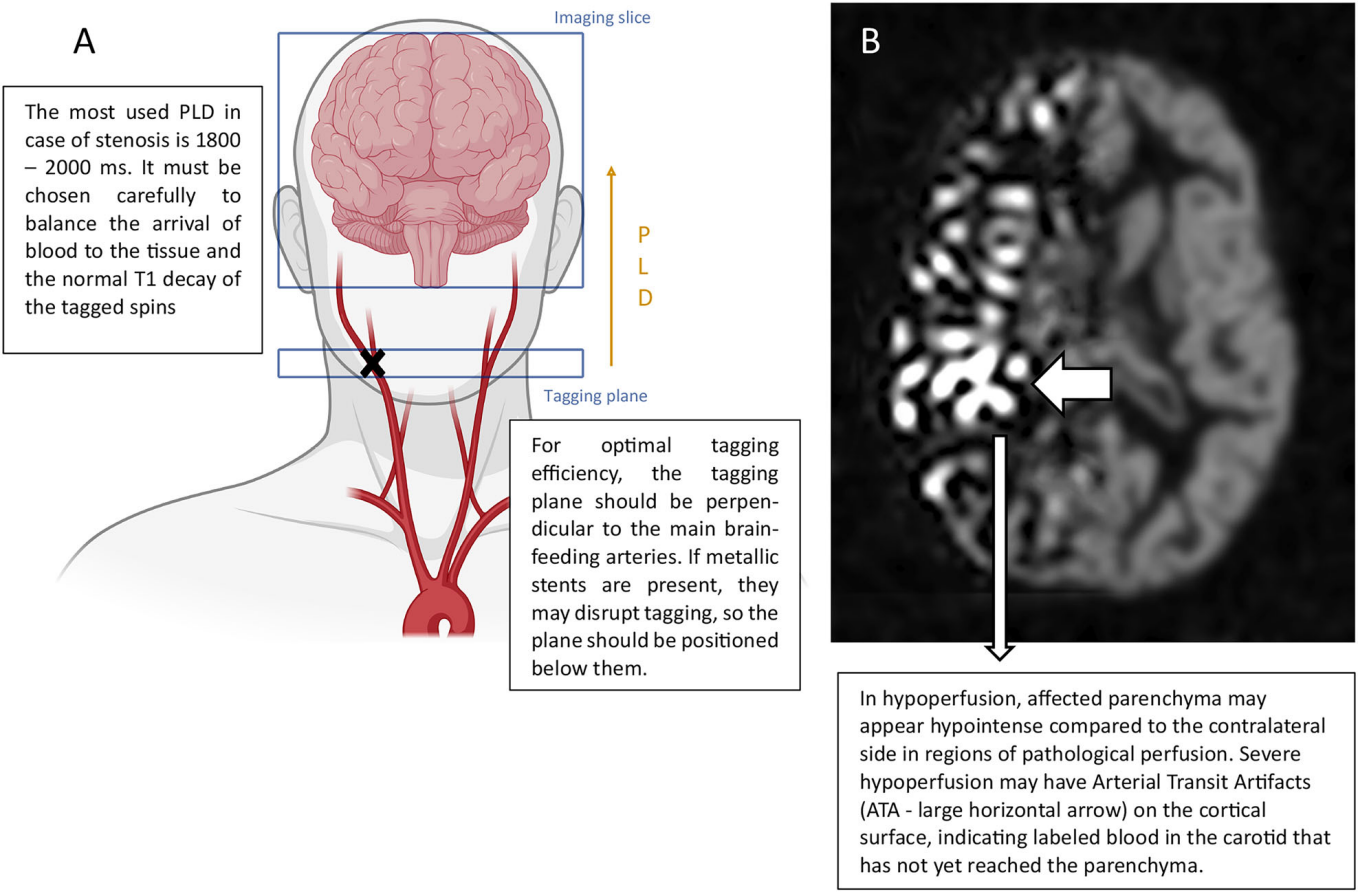
In ASL, selective radiofrequency pulses tag arterial blood, typically at the neck, to track its flow into brain tissue (**A**). In hypoperfusion, affected parenchyma appears hypointense. Arterial transit artifacts (ATA) may appear as hyperintensities on the brain surface due to tagged blood remaining in pial arteries, caused by slow flow or enhanced collateral circulation (**B**)

Among these challenges, arterial transit artifacts (ATAs) are notable. These appear as oval, round, or serpiginous hyperintensities observed on the surface of the brain parenchyma, resulting from tagged blood remaining in the pial arteries within the subarachnoid space (Fig. 1). This occurs either due to slower blood flow, preventing the blood from reaching the parenchyma by the time the signal is acquired, or due to enhanced collateral circulation, where slower-moving blood persists in the pial arteries.

ATA has the potential to serve as purely visual markers of impaired perfusion [7]. However, their full diagnostic significance remains to be elucidated.

This study aimed to systematically review the literature to evaluate the potential diagnostic and therapeutic impact of CBF changes measured using ASL perfusion in patients with extracranial carotid stenosis or occlusion undergoing revascularization procedures.

## Materials and methods

The study was conducted following the Preferred Reporting Items for Systematic Reviews and Meta-Analyses (PRISMA) (PROSPERO registration with the ID number CRD420250656416) guidelines to assess the clinical impact of CBF changes measured by ASL perfusion MRI in patients with carotid artery stenosis undergoing revascularization and to explore the potential implications of ASL for clinical practice.

### Inclusion and exclusion criteria

We included studies focusing on human patients with atherosclerotic stenosis or occlusion of the extracranial internal carotid artery who underwent carotid revascularization and ASL MRI.

We excluded studies involving animals, healthy volunteers, patients with stenosis in blood vessels other than the internal carotid artery, or those with non-atherosclerotic vascular diseases (e.g., Moyamoya disease).

### Search strategy

**Fig. 2:**
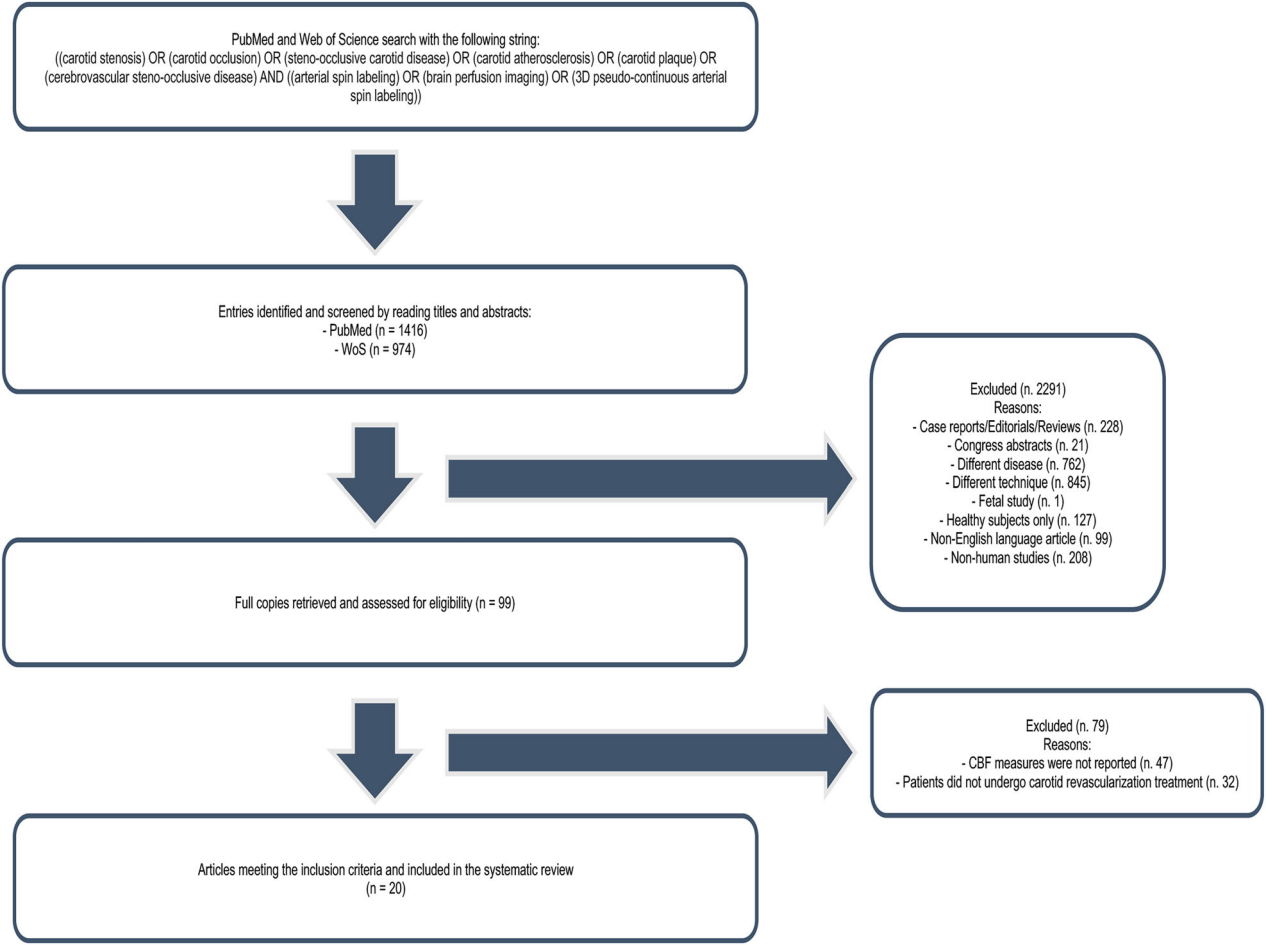
Studies inclusion flowchart

A search was conducted in the MEDLINE/PubMed and Web of Science databases using the following strategy: ((carotid stenosis) OR (carotid occlusion) OR (steno-occlusive carotid disease) OR (carotid atherosclerosis) OR (carotid plaque) OR (cerebrovascular steno-occlusive disease) AND ((arterial spin labeling) OR (brain perfusion imaging) OR (3D pseudo-continuous arterial spin labeling)). Only original articles published in English in international peer-reviewed journals up to May 2nd, 2024, were included. Figure 2 illustrates the study selection process.

### Selection process

All entries retrieved from the two web databases were processed independently by two authors (A.C. and L.L.G.). First, preliminary sources were pooled, and duplicates were removed. The selection process was then carried out in two steps.

Initially, the articles’ titles and abstracts were reviewed to determine whether or not they met the inclusion criteria. Articles that could not be immediately discarded were then read in full to assess their eligibility.

### Data extraction and statistical analysis

Five authors (A.C., L.L.G., C.G., E.M., and L.V.P.) independently reviewed all eligible entries in full, including figures, tables, and supplementary materials. They recorded details on participant conditions, including the degree of stenosis, the number of symptomatic or asymptomatic patients, and the treatments received. They also noted demographic information (age and sex), ASL acquisition parameters (magnetic field strength, sequence type, repetition time, echo time, voxel size, no. of slices, slice thickness, field of view, labeling duration, PLD, and acquisition time). Additionally, perfusion processing details were analyzed, including the software used and the approach for extracting measurements. A list of all numerical variables employed in each study, along with a brief summary of each study’s findings as recorded. This analysis was conducted using Microsoft Excel spreadsheets.

Numerical values are presented as mean ± standard deviation or median ± interquartile range, depending on the format reported in the original study. Results of correlation and statistical significance tests are included where available. For CBF measurements, tables were used to summarize the measurements due to the heterogeneity in measurement strategies across studies. Whenever possible, measurements for the entire internal carotid artery (ICA) territory were provided.

### Risk of bias

An important source of bias is the heterogeneity in ASL MRI acquisition and post-processing parameters across the reported studies. Consequently, the results in terms of CBF (mL/100 g/min) cannot be directly compared or analyzed homogeneously. Instead, CBF changes before and after revascularization are only summarized narratively. A formal meta-analysis cannot be performed.

Additional sources of bias include variability in scanner models (1.5 T vs. 3.0 T), timing of revascularization relative to imaging, and the proportion of symptomatic vs. asymptomatic patients across studies, which may influence perfusion outcomes. Lastly, many of the included studies had a small sample size cohort and were single-center study designs.

## Results

After screening abstracts and titles, 99 studies were reviewed in full. Of these, 20 studies met all inclusion criteria and were included in the final review.

### Study population

Twenty studies [3, 4, 8–25] focused on 710 patients (122 females, age 66.8 ± 6.9 years, range 46–96 years) who underwent carotid revascularization. Specifically, 518 patients (73.0%) underwent CEA, 186 patients (26.2%) underwent CAS, and 6 patients (0.8%) underwent extra-intracranial bypass (ECIC). Among these patients, 234 (33.0%) had asymptomatic stenosis, while 241 (33.9%) were symptomatic in the 6 months before the investigation. The symptomatic status of the remaining 235 patients was not reported.

The percentage of stenosis was assessed using carotid artery Doppler ultrasound (US), MR angiography (MRA), CT angiography (CTA), or digital subtraction angiography (DSA).

Supplementary Material Table 1 provides a more comprehensive overview of the demographic parameters of these studies.

### Acquisition and post-processing

Five studies [3, 8, 10, 13, 23] employed 1.5 T MRI systems, while the remaining fifteen [4, 9, 11, 12, 14–22, 24, 25] 3.0 T MRI systems.

Regarding the type of ASL, three studies [3, 13, 20] used PASL, one study used CASL [8], and sixteen studies [4, 10–12, 14–19, 21–25] used pCASL. Four studies [3, 4, 8, 13] used 2D acquisitions, while ten studies [9, 12, 14–16, 18, 21, 22, 24, 25] employed 3D sequences. In six studies, this data was not reported.

Ten studies [3, 4, 8, 9, 12, 14, 17, 19, 23, 25] used single PLD acquisition, with a median PLD of 1800 ms (range 1500–2500 ms), while seven studies [10, 11, 13, 16, 18, 22, 24] employed multi PLD acquisitions with the number of PLD ranging from 2 to 10 (PLD range: 300–3000 ms). For three studies this data was not reported.

Two studies [3, 4] employed vessel-encoded ASL (VE-ASL) with separate tagging for both ICAs, both external carotid arteries (ECAs), and the posterior circulation.

Supplementary Material Table 2 provides a more comprehensive overview of the studies’ acquisition parameters.

### ASL analysis methods and results

#### Advanced Post Processing software

A total of 13 studies used advanced computational techniques to calculate changes in CBF.

Specifically, 8 studies used manual regions of interest (ROIs) drawn over the CBF maps by an expert neuroradiologist, while the remaining 5 studies used automatic ROI selection strategies, primarily based on MNI atlas segmentation (4 studies).

All these studies [3, 4, 8, 10, 13, 15, 16, 18–20, 22, 23] observed a perfusion deficit on the side ipsilateral to the stenosis before treatment (e.g., ref. [13], Ipsilateral hemisphere: 53.7 ± 14.4 mL/100 g/min, contralateral hemisphere: 64.3 ± 18.5 mL/100 g/min, detailed results are available in Table 1), with deficits being more pronounced in the border zone territories [18]. Following revascularization procedures, CBF values increased (e.g., ref. [9], +13.2 mL/100 g/min, +19.9%), particularly in cases with a greater initial perfusion deficit. The improvement was most notable in the eloquent areas of the anterior circulation territory [8, 23]. Less pronounced increases were also observed in the same areas contralateral to the stenosis [13, 21]. A reduced post-treatment CBF increase was significantly associated with higher systolic blood pressure (*p* = 0.0036), chronic renal insufficiency (*p* = 0.0007), and previous stroke (*p *= 0.0002) [19].

**Table 1:** Summary of CBF measurements in studies that performed a comparison before/after revascularization treatment

Article	Journal	No. of patients	No of healthy controls	preoperative CBF (mL/100 g/min)	postoperative CBF (mL/100 g/min)
Lindner et al [16]	Magn Reson Imaging	17 (5 females)	0	Ipsilateral: 39.07 ± 23.37, Contralateral: 42.45 ± 24.50	Ipsilateral: 44.82 ± 25.91, Contralateral: 42.89 ± 23.61
Xu et al [22]	Front Neurosci	24 (3 females)	0	n.r. in plain text	n.r. in plain text
Fan et al [11]	J Magn Reson Imaging	79 (16 females)	0	Ipsilateral MCA territory hyperperfusion: 24.99 ± 4.67, normal pts: 33.96 ± 6.92	n.r. in plain text
Liu et al [17]	Front Neurol	61 (6 females)	0	n.r. in plain text	n.r. in plain text
Fan et al [12]	Front Cardiovasc Med	77 (19 females)	0	n.r. in plain text	n.r. in plain text
Wang et al [21]	Sci Rep	24 (4 females)	0	Frontoparietal areas. 39.10 ± 4.31 ipsilateral, 44.08 ± 4.68 contralateral.	Day 1. Frontoparietal. 46.93 ± 7.88 ipsilateral, 45.69 ± 4.85 contralateral. Day 2. Frontoparietal. 48.23 ± 9.07 ipsilateral, 47.89 ± 6.28 contralateral. Day 3. Frontoparietal. 50.49 ± 8.02 ipsilateral, 50.16 ± 5.79 contralateral. Day 4. Frontoparietal. 45.58 ± 9.37 ipsilateral, 45.48 ± 6.50 contralateral.
Endo et al [10]	Neurol Res	61 (7 females)	0	n.r. in plain text	n.r. in plain text
Soman et al [19]	J Magn Reson Imaging	53 (0 females)	0	n.r. in plain text	n.r. in plain text
Schröder et al [18]	NeuroImage Clinical	17 (5 females)	0	n.r. in plain text	n.r. in plain text
Lan et al [14]	Radiol Med	32 (4 females)	0	Ipsilateral ICA territory. CAS: 40.21 ± 5.10, CEA: 44.68 ± 8.20 Contralateral ICA territory. CAS: 43.56 ± 7.05, CEA: 49.35 ± 8.77	Day 1. Ipsilateral ICA territory. CAS: 49.02 ± 7.62, CEA: 59.88 ± 8.61. Contralateral ICA territory. CAS: 48.30 ± 8.58, CEA: 59.61 ± 6.25. Day 2. Ipsilateral ICA territory. CAS: 47.72 ± 12.68, CEA: 62.08 ± 11.01. Contralateral ICA territory. CAS: 48.90 ± 11.51, CEA: 60.34 ± 11.48. Day 3. Ipsilateral ICA territory. CAS: 49.12 ± 10.12, CEA: 57.93 ± 10.79. Contralateral ICA territory. CAS: 49.76 ± 9.73, CEA: 59.16 ± 10.46. Day 4. Ipsilateral ICA territory. CAS: 45.09 ± 8.55, CEA: 58.84 ± 8.76. Contralateral ICA territory. CAS: 47.08 ± 10.06, CEA: 60.10 ± 7.80.
Lin et al [15]	Eur Rad	48 (12 females)	0	n.r. in plain text	n.r. in plain text
Wang et al [20]	Front Neurol	16 (4 females)	0	n.r. in plain text	n.r. in plain text
Chen et al [9]	Clin Radiol	25	0	Ipsilateral: 44,4 ± 9,8, Contralateral: 46,5 ± 5,7	Ipsilateral ICA territory: 57,6 ± 8,6, Contralateral 52,7 ± 4,0
Yun et al [23]	Neuroradiology	20 (3 females)	0	Ipsilateral: 29.31 ± 7.48, Contralateral: 30.76 ± 8.25	Ipsilateral: 33.89 ± 8.16, Contralateral: 33.33 ± 7.50
Dang et al [4]	AJNR	8 (3 females)	12 (6 females)	n.r. in plain text	n.r. in plain text
Van Laar et al [3]	J. Vasc. Surg.	24 (9 females)	40 (15 females)	n.r. in plain text	n.r. in plain text
Jones et al [13]	NMR Biomed	20 (8 females)	0	MCA territory. Ipsilateral: 53.7 ± 14.4, contralateral: 64.3 ± 18.5	MCA territory. Ipsilateral: 58.5 ± 11.6, contralateral: 67.3 ± 18.0
Ances et al [8]	J Neuroimaging	10 (3 females)	0	Ipsilateral ICA territory: 41.8 ± 4.6	Ipsilateral ICA territory: 42.7 ± 3.6

*CBF* cerebral blood flow, *MCA* middle cerebral artery, *CAS* carotid artery stenting, *CEA* carotid endarterectomy, *n.r*. not reported. When non-otherly specified, numerical measures refer to the whole internal carotid artery vascular territory

Two studies [18, 20] investigated the relationship between perfusion and cognitive performance outcomes in patients with asymptomatic carotid artery stenosis undergoing revascularization procedures. No significant association was found between cognitive test results and either preoperative hypoperfusion [18] or increased CBF after CAS [20].

Regarding postoperative cerebral hyperperfusion (CH), Lin et al [15] reported that patients with post-operative CH exhibited a significantly higher spatial coefficient of variation of CBF (CoV CBF: 0.35 CH vs. 0.28 non-CH, *p* = 0.007) and a higher ratio of the volume perfused by the stenotic carotid artery pre-intervention to the total brain volume (RatioPV: 0.92 CH vs. 0.96 non-CH, *p* = 0.012) compared to patients without CH. Preoperative MRA also showed a higher prevalence of type P Circle of Willis, i.e., with hypo/aplastic anterior communicating artery (AcomA) or A1 segment of the anterior cerebral artery (ACA), in patients with CH [15] (100% vs. 35.7%).

Endo et al [10] used multi-PLD ASL to analyze post-CEA CH. They calculated the asymmetry index (AI), defined as the ratio of CBF between the affected and unaffected sides in the (MCA) territory. A linear regression analysis was conducted to assess the relationship between AI values derived from ASL at three different PLDs (1525, 2025, and 2525 ms). From this analysis, a slope index was obtained, theoretically reflecting differences in arterial transit time between the affected and unaffected sides and serving as a surrogate marker for cerebrovascular reactivity disparities. The slope index proved to be a reliable predictor of CH, with 85% sensitivity and 74% specificity.

Two studies investigated VE-ASL changes in patients undergoing revascularization.

One study [4] compared the effectiveness of VE-ASL and DSA in assessing collateral flow in carotid stenosis patients undergoing CAS. It demonstrated that VE-ASL could detect reduced ipsilateral ICA flow due to stenosis, accompanied by increased compensatory perfusion from the contralateral ICA and vertebral artery territories, which normalized after surgery. Van Laar et al [3] also observed smaller ICA territories with larger contralateral and vertebral artery territories before revascularization, which normalized after both CEA and CAS, with no significant differences between the two procedures.

*Findings in symptomatic patients*:

- A significantly smaller increase in CBF after treatment compared to asymptomatic patients was found [19].
- VE-ASL successfully detected preoperative collateral flow changes and normalization after revascularization, showing similar findings across CEA and CAS procedures [3, 4].

*Findings in an asymptomatic patient*:

- No significant association was found between CBF variations pre/post-treatment and cognitive performance [18, 20].

*Findings in both symptomatic and asymptomatic patients*:

- Reduced CBF was found on the hemisphere ipsilateral to the stenosis before treatment, compared to the contralateral or to healthy controls, especially on the border between vascular territories [3, 4, 8, 10, 13, 15, 16, 18–20, 22, 23].
- Revascularization led to increased CBF, particularly in the anterior circulation and in patients with greater baseline deficits [3, 4, 8, 10, 13, 15, 16, 18–20, 22, 23].
- High systolic BP and renal insufficiency were associated with smaller CBF improvements after treatment [19].
- Patients who developed postoperative CH had higher spatial CBF variation and more often had an incomplete (type P) Circle of Willis [15].
- The preoperative asymmetry of the ratio of CBF between the affected and unaffected sides in the MCA territory can reliably predict postoperative CH [10].

#### Purely visual strategies

Three studies used purely visual strategies to evaluate ASL images. One study [11] assessed perfusion using a four-point “ASL score” based on signal intensity from the parenchyma in a three-PLD ASL sequence: (i) Poor: impaired perfusion in all three phases, (ii) Intermediate: reduced perfusion area and/or signal in the early and middle phases, with normal perfusion in the late phase, (iii) Good: reduced perfusion area and/or signal in the early phase, with normal perfusion in the middle and late phases, (iv) Excellent: normal perfusion. A poor ASL perfusion score was predictive of post-revascularization CH (OR = 37.33, 95% CI (4.75, 293.63)), as well as higher baseline blood pressure (OR = 1.08, 95% CI (1.02, 1.14)), and carotid near-occlusion (OR = 7.31, 95% CI (1.16, 46.06)).

Fan et al [25] visually assessed perfusion in the MCA territory using a standardized scoring system based on the presence of ATA and the signal intensity from the parenchyma on a mono PLD sequence. Each of the 10 locations included in the Alberta Stroke Program Early CT Score (ASPECTS) was assigned a score based on the following scale: 0 (no/minimal ASL signal), 1 (low/moderate ASL signal with ATA), 2 (high ASL signal with ATA), 3 (high ASL signal without ATA). The final score was calculated as the sum of the points assigned to each location

The preoperative ASL score was an independent predictor of CH (OR = 0.48 (95% CI 0.33–0.71), *p* < 0.001), with an optimal cutoff value of 25 points (AUC = 0.98, 94.1% sensitivity, 88.4% specificity).

The remaining study [24] visually analyzed subtraction images created from two different PLDs in patients who underwent CEA.

This method helped differentiate the persistence of high signal intensities due to ATA from the abrupt CBF increases associated with CH in post-operative ASL, as the ATA, being present in both images, is canceled out.

*Findings in both symptomatic and asymptomatic patients*:

- ASL images suggestive of poor perfusion (i.e., low parenchymal signal associated with ATA presence) are associated with a higher risk of developing postoperative CH [11, 25].
- Subtraction images from two PLDs can visually distinguish true hyperperfusion from ATA in post-operative ASL, improving the interpretation of signal changes after surgery [24].

#### MRI workstation software

A study [9] focused on the changes in CBF after CAS, reporting a significant post-procedural increase in CBF (44.4 ± 9.8 vs. 57.6 ± 8.6, +58.6% increase). However, patients with high stenting positions showed lower post-CAS CBF values (31.0 ± 4.8, –28.9%) due to distortion artifacts in the labeling plane that interfere with the tagging process. Indeed, this false hypoperfusion normalized after repeating the scan with the tagging plane positioned under the stent.

Another study [12] focused on postoperative CH, found that white matter hyperintensities (WMH) and lacunes were independently associated with postoperative CH. Receiver operating characteristic (ROC) curve analysis identified a Fazekas score (sum of deep and periventricular white matter) ≥ 3 and the presence of at least 2 lacunes as optimal predictors for CH (AUC = 0.84 and 0.73, respectively).

The remaining study [17] combined ASL with carotid vessel-wall imaging, revealing that carotid intraplaque hemorrhage (IPH) was associated with a more pronounced preoperative reduction in CBF (*r* = −0.127, *p* = 0.012). Additionally, larger IPH size was also associated with a smaller increase in CBF after CEA (*r *= −0.060, *p* = 0.020).

Two further studies initially processed ASL perfusion data on an MRI workstation (all employing GE workstation tools) before transferring the images to a separate computer for further advanced analysis.

These studies examined CBF changes over time in the immediate postoperative period. Lan et al [14] observed that the CBF increased postoperatively, peaking at different times between CAS (maximum at 48 h post-surgery, 49.12 ± 10.12 mL/100 g/min) and CEA (maximum at 72 h post-surgery, 58.84 ± 8.76 mL/100 g/min).

Wang et al [21] reported similar post-CAS results, noting a greater CBF increase in the ipsilateral frontal and parietal lobes compared to the contralateral sides (CBF increase at day 3, Ipsilateral: 11.39 ± 9.10 mL/100 g/min, contralateral: 6.08 ± 7.45 mL/100 g/min). Furthermore, patients with new diffusion-weighted imaging (DWI) lesions on postoperative MRI, suggestive of microembolism, had significantly lower relative CBF in the frontal and parietal lobes compared to those without microembolism (0.861 vs. 0.912, *p * < 0.05).

*Findings in symptomatic patients*:

- Peak CBF increase occurred at different times between CAS (48 h post-treatment) and CEA (72 h) [14].

*Findings in both symptomatic and asymptomatic patients*:

- High stenting positions might lead to artificially low CBF readings due to labeling artifacts, which normalized after lowering the tagging plane under the stent [9].
- White matter hyperintensities and lacunes were independent predictors of postoperative CH. A Fazekas score ≥ 3 and ≥ 2 lacunes showed good predictive value (AUC = 0.84 and 0.73, respectively) [12].
- Presence and large size of IPH correlated with lower preoperative CBF and smaller postoperative CBF increases [17].
- CBF increase after CAS was significantly lower in patients with new diffusion-weighted imaging (DWI) lesions suggestive of microembolism [21].”

Supplementary Material Table 3 provides a more comprehensive overview of these studies’ analysis strategies and findings.

## Discussion

The results of this systematic review highlight the value of ASL MRI in evaluating cerebral perfusion changes in patients with carotid artery stenosis or occlusion undergoing revascularization procedures. ASL effectively detects pre-treatment ipsilateral perfusion deficits and post-treatment improvements, particularly in eloquent regions of the anterior circulation anterior circulation [3, 4, 8–23]. Interestingly, certain cardiovascular risk factors, i.e., hypertension, chronic renal insufficiency, and previous stroke, are significantly associated with a reduced post-procedural CBF increase following CAS and CEA [19]. Moreover, patients who developed microembolism during the revascularization procedure, as detected by post-procedural diffusion-weighted imaging, in the territory of the stenotic carotid artery had lower post-CAS treatment increases in relative cerebral blood flow [21], suggesting that procedural complications may impact treatment outcomes.

ASL could play a pivotal role in three key clinical scenarios: (1) assessing the risk of cerebral hyperperfusion syndrome, (2) differentiating cerebral hemodynamic patterns in symptomatic vs. asymptomatic patients, and (3) evaluating collateral blood flow in patients with carotid occlusion.

### Cerebral hyperperfusion syndrome risk assessment

We observed that ASL can help identify patients at higher risk of cerebral hyperperfusion syndrome. One interesting factor associated with CH is a higher pre-intervention ratio of the volume perfused by the stenotic carotid artery to the total brain volume [15]. The authors suggest that a higher proportion of cerebral tissue perfused by the stenotic artery indicates an impaired capacity to recruit blood flow from collateral vessels via the circle of Willis. This limited ability to establish collateral circulation in the presence of stenosis suggests a reduced capacity to adapt to sudden increases in perfusion following reperfusion treatment [26]. Consequently, this may contribute to the development of post-procedural hyperperfusion. Additionally, anatomical variations in the circle of Willis—such as the absence or hypoplasia of the AcomA or A1 segment [15]—were significant risk factors for CH, highlighting the role of impaired blood flow autoregulation in its development. Other risk factors, all indicative of severe hemodynamic compromise, include small vessel disease features (e.g., WMH and lacunes) [12], hypertension, and carotid near-occlusion [11].

Moreover, ASL-derived metrics, including ATA [11, 25] and the CBF ratio between ipsilateral and contralateral hemispheres [15], have proven useful in predicting CH. For instance, visual assessments of preoperative ASL images have effectively identified patients at risk of CH, offering a quick, minimal post-processing alternative [11, 25]. While Doppler US remains valuable for assessing flow velocities in the MCA, it does not provide direct perfusion measurements of the parenchyma, highlighting the added value of ASL in this context. Moreover, Doppler values are typically collected postoperatively to monitor patients, but they cannot identify those at risk of postoperative syndrome beforehand.

Despite these advantages, the lack of a standardized definition of CH complicates comparisons across studies. Current definitions vary, from a relative post-procedural CBF increase of over 100% [11, 25] to absolute CBF exceeding the contralateral side [15]. Establishing standardized criteria is crucial for broader clinical adoption of ASL in CH risk assessment.

### Differences between symptomatic and asymptomatic patients

Patients with a history of stroke have been observed to exhibit a smaller increase in CBF six months after CEA or CAS. This phenomenon appears to be related to differences in the physiological response of cerebral arteries that occur in patients with cerebral ischemia and persist over time [19]. These findings highlight that the same treatment strategy may not yield uniform outcomes across all individuals, reinforcing the growing consensus that managing carotid artery stenosis requires a personalized approach.

### Assessing collateral blood flow

ASL can assess the extent of compensatory perfusion through collateral pathways, including both primary collaterals through the circle of Willis and secondary collaterals from the external carotid arteries (ECA), using a specialized technique capable of assessing the vascular territory of each vessel called VE-ASL [3, 4]. This technique may be particularly valuable in patients with asymptomatic carotid occlusion. Indeed, an increase in perfusion from the ECA in these patients was found compared to healthy volunteers that normalizes following revascularization [4]. This underscores the ECA’s critical role in cerebral perfusion and suggests that this method could help guide revascularization strategies.

### Technical considerations

Understanding key technical aspects of ASL acquisition can enhance its clinical utility in patients with carotid stenosis.

Firstly, stent placement can interfere with CBF measurements in CAS patients. High-positioned stents at the C2-C3 level, where the labeling plane is typically placed, often result in false postoperative hypoperfusion caused by distortion artifact from the metal in the stent that interferes with the tagging of the blood protons. Indeed, this artifact disappears when the labeling plane is moved under the stent [9].

Optimal PLD selection is crucial for accurate CBF quantification, particularly in patients with prolonged arterial transit times, such as those with carotid stenosis. An insufficient PLD may underestimate CBF, while an excessively long PLD can lead to signal loss due to T_1_ decay [27]. Multi-PLD ASL can account for transit time variations, though it requires longer scan times and greater complexity. Visual assessment methods, such as dual-PLD ASL, have shown promise in differentiating ATA signals from true hyperperfusion in post-operative ASL [24], highlighting the need for further exploration of multi-PLD approaches in clinical practice.

Interestingly, ASL has also revealed differences in CBF dynamics following different revascularization procedures. Postoperative CBF increases peak at different times: 48 h after CAS and 72 h after CEA, with more pronounced increases in the frontal and parietal lobes ipsilateral to the stenosis [14, 21]. These findings suggest that post-procedural monitoring protocols may need to be tailored to account for different high-risk CH time windows. However, these differences diminish over time, with no significant regional CBF disparities between CAS and CEA patients at one-month post-procedure—aligning with values observed in healthy individuals [3].

### Limitations

This systematic review presents some limitations that should be considered when interpreting the results.

Firstly, significant heterogeneity was present in the different ASL protocols across the included studies.

The included studies demonstrated consistent trends, mainly the ability to detect perfusion deficits and hyperperfusion risk, as well as the ability to assess collaterals. However, substantial variability in ASL protocols and patient populations limits direct comparability.

ASL suffers from substantial variability in acquisition parameters, including labeling strategies, PLDs, and readout schemes, as well as in post-processing methodologies. These inconsistencies complicate direct comparisons between studies and may affect the robustness of the conclusions. Additionally, ASL has an inherently low signal-to-noise ratio, making image processing and quantitative analysis challenging. The relatively weak perfusion signal and physiological and technical noise further contribute to the intrinsic variability of ASL-based CBF quantification. Absolute perfusion values are also influenced by multiple factors such as age, sex, circulatory velocity, blood pressure, drugs or vasoactive substances (e.g., caffeine), sleep/wake cycle, mood, and heart failure. Overall, variability in CBF measurements remains a significant concern, influenced by the factors mentioned. Inter-session variability in individual CBF measurements can be around 5%, with variations over weeks reaching 10–20% [28].

Additional sources of bias include variability in scanner models (1.5 T vs. 3.0 T), timing of revascularization relative to imaging, and the proportion of symptomatic vs. asymptomatic patients across studies, which may influence the perfusion outcomes of our results.

Second, there was limited use of some specific techniques, such as the VE-ASL, which allows selective assessment of perfusion in specific vascular territories targeted, mainly due to the lack of standardized implementation of this sequence across MRI vendors, as well as the need for operator expertise to select the appropriate targeted artery. These factors hinder its routine use, despite its potential value in improving non-invasive evaluation of extracranial and intracranial circulation, linked to technical complexity, lack of standardized protocols, and insufficient clinician familiarity.

Lastly, many of the included studies had a small sample size cohort and were single-center study designs. Overall, these factors may limit the generalizability of the findings to broader and more diverse populations and, while ASL demonstrates promise in the aforementioned specific clinical scenarios, the evidence remains exploratory. Robust validation in prospective cohorts is essential to translate these findings into practice.

### Strength of analysis and synthesis of the results

The strength of our analysis lies in the fact that all studies have demonstrated a similar ability of the sequence to show a reduction in downstream flow distal to the extracranial stenosis. Although the studies were not specifically designed to detect differences between symptomatic and asymptomatic patients, they did not report significant baseline or post-treatment differences between these groups. The most consistently reported finding was that various methods confirmed that a severe reduction in flow, measured as a large hypoperfused hypoperfusion area, as well as the presence of ATA, are both associated with complications such as hyperperfusion syndrome.

### Future directions

Understanding ASL findings in cases of extracranial stenosis provides a valuable foundation for extending its application to intracranial arterial stenosis. As seen in patients with extracranial stenosis, a high volume perfused by the stenotic artery and the severity of hypoperfusion may indicate a hyperperfusion syndrome, which may determine the treatment indication. Current evidence doesn’t support the systematic revascularization of patients with symptomatic intracranial atherosclerotic disease; thus, ASL would add new useful information for clinical practice.

Further studies are needed to evaluate the role of ASL in patients with intracranial stenosis, particularly for characterizing perfusion deficits and guiding therapeutic decisions.

## Conclusions

Our systematic review highlights the ability of ASL to assess cerebral perfusion changes in carotid artery stenosis before and after revascularization treatments. This enables the identification of patients at risk for cerebral hyperperfusion syndrome and allows for the preoperative evaluation of collateral blood flow, thereby potentially improving clinical decision-making.

While ASL shows promise in assessing treatment response and in risk stratification for CH and collateral assessment, further research is needed to determine its role in identifying the risk of future stroke in patients with asymptomatic carotid artery stenosis.

## Supplementary information

Supplementary information

## Abbreviations

AI: Asymmetry index
AcomA: Anterior communicating artery
ASL: Arterial spin labeling
ASL Continuous: Continuous ASL
ASPECTS: Alberta Stroke Program Early CT Score
ATA: Arterial transit artifacts
CAS: Carotid artery stenting
CBF: Cerebral blood flow
CEA: Carotid endarterectomy
CH: Cerebral hyperperfusion
CI: Confidence interval
DSA: Digital subtraction angiography
DWI: Diffusion-weighted imaging
ECA: External carotid arteries
ICA: Internal carotid artery
IPH: Intraplaque hemorrhage
MCA: Middle cerebral artery
MRA: MR angiography
OR: Odds ratio
PASL: Pulsed ASL
pCASL: Pseudo-continuous ASL
PLD: Post-labeling delay
ROIs: Regions of interest
US: Ultrasound
VE-ASL: Vessel-encoded ASL
WMH: White matter hyperintensities

## References

[1] Wintermark M, Sesay M, Barbier E et al (2005) Comparative overview of brain perfusion imaging techniques. Stroke 36:e83–e99 16100027 10.1161/01.STR.0000177884.72657.8b

[2] Petrella JR, Provenzale JM (2000) MR perfusion imaging of the brain: techniques and applications. AJR Am J Roentgenol 175:207–219 10882275 10.2214/ajr.175.1.1750207

[3] Van Laar PJ, Hendrikse J, Mali WP et al (2007) Altered flow territories after carotid stenting and carotid endarterectomy. J Vasc Surg 45:1155–1161 17543680 10.1016/j.jvs.2006.11.067

[4] Dang Y, Wu B, Sun Y et al (2012) Quantitative assessment of external carotid artery territory supply with modified vessel-encoded arterial spin-labeling. AJNR Am J Neuroradiol 33:1380–1386 22345497 10.3174/ajnr.A2978 PMC7965524

[5] Tekieli L, Mazurek A, Dzierwa K et al (2022) Misclassification of carotid stenosis severity with area stenosis-based evaluation by computed tomography angiography: impact on erroneous indication to revascularization or patient (lesion) migration to a higher guideline recommendation class as per ESC/ESVS/ESO/SVS and CMS-FDA thresholds. Postepy Kardiol Interwencyjnej 18:500–513 36967857 10.5114/aic.2023.125610 PMC10031677

[6] Bagley JH, Priest R (2020) Carotid revascularization: current practice and future directions. Semin Intervent Radiol 37:132–139 32419725 10.1055/s-0040-1709154 PMC7224973

[7] Di Napoli A, Cheng SF, Gregson J et al (2020) Arterial spin labeling MRI in carotid stenosis: arterial transit artifacts may predict symptoms. Radiology 297:652–660 33048034 10.1148/radiol.2020200225

[8] Ances BM, McGarvey ML, Abrahams JM et al (2004) Continuous arterial spin labeled perfusion magnetic resonance imaging in patients before and after carotid endarterectomy. J Neuroimaging 14:133–138 15095558

[9] Chen DY, Kuo YS, Hsu HL et al (2016) Loss of labelling efficiency caused by carotid stent in pseudocontinuous arterial spin labelling perfusion study. Clin Radiol 71:e21–e27 26620708 10.1016/j.crad.2015.10.004

[10] Endo H, Fujimura M, Saito A, Endo T, Ootomo K, Tominaga T (2021) Efficacy of arterial spin labeling magnetic resonance imaging with multiple post-labeling delays to predict postoperative cerebral hyperperfusion in carotid endarterectomy. Neurol Res 43:252–258 33190623 10.1080/01616412.2020.1847529

[11] Fan X, Lai Z, Lin T et al (2023) Multidelay MR arterial spin labeling perfusion map for the prediction of cerebral hyperperfusion after carotid endarterectomy. J Magn Reson Imaging 58:1245–1255 36951494 10.1002/jmri.28634

[12] Fan X, Lai Z, Lin T et al (2021) Pre-operative cerebral small vessel disease on mr imaging is associated with cerebral hyperperfusion after carotid endarterectomy. Front Cardiovasc Med 8:734392 34869635 10.3389/fcvm.2021.734392 PMC8636731

[13] Jones CE, Wolf RL, Detre JA et al (2006) Structural MRI of carotid artery atherosclerotic lesion burden and characterization of hemispheric cerebral blood flow before and after carotid endarterectomy. NMR Biomed 19:198–208 16475206 10.1002/nbm.1017

[14] Lan Y, Lyu J, Ma X, Ma L, Lou X (2019) Longitudinal assessment of cerebral blood flow changes following carotid artery stenting and endarterectomy. Radiol Med 124:636–642 30771219 10.1007/s11547-018-00986-7

[15] Lin T, Lai Z, Zuo Z et al (2019) ASL perfusion features and type of circle of Willis as imaging markers for cerebral hyperperfusion after carotid revascularization: a preliminary study. Eur Radiol 29:2651–2658 30443757 10.1007/s00330-018-5816-1

[16] Lindner T, Cheng B, Heinze M et al (2024) A comparative study of multi and single post labeling delay pseudocontinuous arterial spin labeling in patients with carotid artery stenosis. Magn Reson Imaging 106:18–23 38042453 10.1016/j.mri.2023.11.011

[17] Liu Y, Huo R, Xu H et al (2022) Associations between carotid plaque characteristics and perioperative cerebral blood flow determined by arterial spin labeling imaging in patients with moderate-to-severe stenosis undergoing carotid endarterectomy. Front Neurol 13:899957 35865645 10.3389/fneur.2022.899957 PMC9295123

[18] Schroder J, Heinze M, Gunther M et al (2019) Dynamics of brain perfusion and cognitive performance in revascularization of carotid artery stenosis. Neuroimage Clin 22:101779 30903966 10.1016/j.nicl.2019.101779 PMC6431743

[19] Soman S, Dai W, Dong L et al (2020) Identifying cardiovascular risk factors that impact cerebrovascular reactivity: An ASL MRI study. J Magn Reson Imaging 51:734–747 31294898 10.1002/jmri.26862 PMC6954347

[20] Wang T, Sun D, Liu Y et al (2017) The impact of carotid artery stenting on cerebral perfusion, functional connectivity, and cognition in severe asymptomatic carotid stenosis patients. Front Neurol 8:403 28848495 10.3389/fneur.2017.00403 PMC5552726

[21] Wang WX, Wang T, Ma L, Sun ZH, Wang GS (2021) New-onset lesions on MRI-DWI and cerebral blood flow changes on 3D-pCASL after carotid artery stenting. Sci Rep 11:8005 33850199 10.1038/s41598-021-87339-z PMC8044121

[22] Xu H, Han H, Liu Y et al (2023) Perioperative cerebral blood flow measured by arterial spin labeling with different postlabeling delay in patients undergoing carotid endarterectomy: a comparison study with CT perfusion. Front Neurosci 17:1200273 37781254 10.3389/fnins.2023.1200273 PMC10536277

[23] Yun TJ, Sohn CH, Han MH et al (2013) Effect of carotid artery stenting on cerebral blood flow: evaluation of hemodynamic changes using arterial spin labeling. Neuroradiology 55:271–281 23093072 10.1007/s00234-012-1104-y

[24] Haga S, Morioka T, Kameda K et al (2019) Subtraction of arterial spin-labeling magnetic resonance perfusion images acquired at dual post-labeling delay: Potential for evaluating cerebral hyperperfusion syndrome following carotid endarterectomy. J Clin Neurosci 63:77–83 30738738 10.1016/j.jocn.2019.01.044

[25] Fan X, Zuo Z, Lin T et al (2022) Arterial transit artifacts on arterial spin labeling MRI can predict cerebral hyperperfusion after carotid endarterectomy: an initial study. Eur Radiol 32:6145–6157 35394182 10.1007/s00330-022-08755-x

[26] Ogasawara K, Yukawa H, Kobayashi M et al (2003) Prediction and monitoring of cerebral hyperperfusion after carotid endarterectomy by using single-photon emission computerized tomography scanning. J Neurosurg 99:504–510 12959438 10.3171/jns.2003.99.3.0504

[27] Alsop DC, Detre JA, Golay X et al (2015) Recommended implementation of arterial spin-labeled perfusion MRI for clinical applications: A consensus of the ISMRM perfusion study group and the European consortium for ASL in dementia. Magn Reson Med 73:102–116 24715426 10.1002/mrm.25197 PMC4190138

[28] Clement P, Mutsaerts HJ, Vaclavu L et al (2018) Variability of physiological brain perfusion in healthy subjects—a systematic review of modifiers. Considerations for multi-center ASL studies. J Cereb Blood Flow Metab 38:1418–1437 28393659 10.1177/0271678X17702156 PMC6120130

