# Supplementary material for: Arterial spin labeling MRI in patients undergoing carotid artery revascularization: a systematic review of the hemodynamic changes and clinical implications
Source: Eur Radiol. 2025 Aug 6;36(2):916–27. doi: 10.1007/s00330-025-11885-7 (PMC12953264; doi:10.1007/s00330-025-11885-7)
Supplement: Supplementary file 1 — Supplementary information [file 330_2025_11885_MOESM1_ESM.pdf]

Arterial Spin Labeling MRI in Patients Undergoing Carotid Artery Revascularization: a Systematic Review of the Hemodynamic Changes and Clinical Implications

ELECTRONIC SUPPLEMENTARY MATERIAL

Eur Radiol (2025) Carrozzi A, Manfini E, Golini C, et al.

Table S1. Summary of patients characteristics of the included studies.

Author (year of publication)	N. of patients	Healthy control	Treatment	Carotid stenosis	Stenosis measurement
**Ances (2004)**	10 (3 females)	0	CEA	- 70-99 %<br>- symptomatic	n.r.
**Jones (2006)**	20 (8 females)	0	17 CEA<br>3 CAS	- 50-99 %<br>- 6 pts. Symptomatic<br>- 14 pts. Asymptomatic	OR (i.e., the ratio of ICA vessel wall area to total vessel area measured on Carotid MRI)
**Van Laar (2007)**	24 (9 females)	40 (15 females)	12 CAS<br>12 CEA	- 50-99 %<br>- symptomatic<br>- unilateral	NASCET on Carotid MRA
**Dang (2012)**	8 (3 females)	12 (6 females)	2 CAS<br>6 ECIC	50-100 %	n.r.
**Yun (2013)**	20 (3 females)	0	CAS	- 50-99 %<br>- 16 pts. Symptomatic<br>- 4 pts. Asymptomatic	n.r.
**Chen (2016)**	25	0	CAS	50-99 %	NASCET
**Wang (2017)**	16 (4 females)	0	CAS	- 70-99 %<br>- unilateral	n.r.
**Haga (2019)**	8 (0)	no	CEA	- 60-73% asymptomatic (3pts)<br>- 58-80% symptomatic (5 pts)	NASCET on Carotid MRA
**Lin (2019)**	48 (12 females)	0	CEA<br>CAS	- 50-99 % if symptomatic<br>- 70-99 % if asymptomatic	NASCET on CTA
**Lan (2019)**	32 (4 females)	0	13 CEA<br>19 CAS	- 70-99 %<br>- symptomatic<br>- unilateral	n.r.
**Schröder (2019)**	17 (5 females)	0	12 TEA<br>5 CAS	- 70-99 %<br>- asymptomatic<br>- unilateral<br>- no history of cognitive impariment	NASCET
**Soman (2020)**	53 (0 females)	0	29 CEA<br>24 CAS	- 60-99 % if symptomatic<br>- 80-99 % if asymptomatic	Carotid Doppler ultrasound
**Endo (2020)**	61 (7 females)	0	CEA	- 50-99 %<br>- 43 pts. Asymptomatic<br>- 18 pts. Symptomatic	NASCET on CTA
**Wang (2021)**	24 (4 females)	0	CAS	- 50-99 %<br>- unilateral<br>- 10 pts. Asymptomatic<br>- 14 pts. Symptomatic	NASCET at Doppler ultrasound, then confirmed by DSA
**Fan (2021)**	77 (19 females)	0	CEA	- 50-99 % if symptomatic (30 pts.)<br>- 70-99 % if asymptomatic (47 pts)<br>- unilateral	NASCET on CTA
**Fan (2022)**	86 (11 females)	0	CEA	- 50-99% if symptomatic (32 pts)<br>- 70-99% if asymptomatic; (54 pts)	NASCET on CTA
**Liu (2022)**	61 (6)	0	CEA	50-99%	CTA
**Fan (2023)**	79 (16 females)	0	CEA	- 50-99 %<br>- unilateral<br>- 27 pts. Asymptomatic<br>- 52 pts. Symptomatic	NASCET on CTA
**Xu (2023)**	24 (3 females)	0	CEA	- 70-99 %<br>- unilarteral	NASCET on CTA
**Lindner (2024)**	17 (5 females)	0	5 CEA<br>12 CAS	- 70-99 %<br>- asymptomatic<br>- unilarteral	NASCET

Legend. CAS: carotid artery stenting; CEA: carotid endarterectomy; pt: patient; NASCET: north american symptomatic carotid endarterectomy trial; MRA: magnetic resonance angiography; CTA: computed tomography angiography.

Table S2. Summary of acquisition and post-processing of the included studies.

Author (year of publication)	MRI sequences	Type of ASL	Readout	Imaging parameters	FOV	Voxel size (mm3)	Labeling duration (ms)	TI or PLD (ms)	Total acquisition time
**Ances (2004)**	CASL	Continuous	Single-shot gradient-echo echo-planar	- Labeling gradient: 0.25 G/cm<br>- Labeling RF: 35 mG<br>- TR: 4000 ms<br>- TE: 22 ms<br>- Slice thickness: 8 mm<br>- Interslice gaps: 2 mm<br>- Acquisition matrix: 64 × 40<br>- Acquisition bandwidth: ± 62.5 kHz	240 x 150 mm	3.75 x 3.75 x 8	n.r.	1500	6 min
**Jones (2006)**	QUIPPS II	Pulsed (modified)	Single-shot echo-planar	- RF amplitude: 22 microT<br>- Tagging efficiency: 95%<br>- TR: 2.3 s<br>- TE: 22 ms<br>- Slice thickness: 7 mm<br>- Spacing between slices: 2.45 mm<br>- Acquisition matrix: 64 × 64<br>- Receiver bandwidth: 128 kHz<br>- Number of averages: 50	240 x 240 mm	3.75 x 3.75 x 7	15.36	- TI1 (delay between the inversion and saturation pulses): 700<br>- TI2 (delay between the inversion and start of image acquisition): 1700 + (n - 1) x 50 ms for the n-th image slice acquired	n.r.
**Van Laar (2007)**	PASL	Pulsed	Single-shot echo-planar	- TR: 3000 ms<br>- TE: 5.6 ms<br>- Partial Fourier acquisition: 62%<br>- Slice thickness: 8 mm<br>- Slice gap: 1 mm<br>- Acquisition matrix: 64 × 64<br>- Zero filling to: 128 × 128 matrix<br>- Number of averages: 30	240 x 240 mm	3.75 x 3.75 x 8	n.r.	1600	9 min
**Dang (2012)**	VE-ASL Hadamard encoded	Pseudo-continuous (modified)	2D single-shot spiral with fat saturation	- Number of RF pulses: 1640<br>- Spacing between RF pulses: 0.96 ms<br>- TR: 3000 ms<br>- TE: 3.4 ms<br>- Section thickness: 8 mm<br>- Section gap: 2 mm<br>- Acquisition matrix: 128 x 128<br>- Number of signal-intensity averages for each cycle: 20	240 x 240 mm	1.88 x 1.88 x 8	1600	1000	12.3 min
**Yun (2013)**	pCASL	Pseudo-continuous	n.r.	- TR: 4525 ms<br>- TE: 9.9 ms<br>- Slice thickness: 5 mm<br>- NEX: 3	240 x 240 mm	5	n.r.	1500	4.8 min
**Chen (2016)**	pCASL	Pseudo-continuous	3D background suppressed fast-spin-echo stack-of-spiral	- TR: 5327 ms<br>- TE: 10.5 ms<br>- No flow-crushing gradients<br>- In-plane matrix: 128 × 128<br>- NEX: 4<br>- Section thickness: 4 mm<br>- Echo train length: 36<br>- Labeling plane: 10 mm thick, placed 2 cm inferior to the lower edge of the cerebellum	640 x 640 mm	4	1500	2525	5.6 min
**Wang (2017)**	Q2TIPS II	Pulsed (modified)	n.r.	- TR: 2500 ms<br>- TE: 11 ms<br>- FA: 90°<br>- Slice thickness: 6 mm	240 x 240 mm	3.75 x 3.75 x 6	n.r.	n.r.	n.r.
**Haga (2019)**	pCASL, Two PLD	Pseudo-continuous	3D spiral fast spin-echo imaging	- TR: 4728 ms<br>- Section thickness. 4mm	n.r.	n.r.	1500	1025, 1525	2,3 min
**Lin (2019)**	pCASL	Pseudo-continuous	3D background suppressed fast-spin-echo stack-of-spiral	n.r.	n.r.	n.r.	n.r.	n.r.	4.7 min
	ss-pCASL	Pseudo-continuous	3D background suppressed fast-spin-echo stack-of-spiral super-selective	n.r.	n.r.	n.r.	n.r.	n.r.	4.3 min
**Lan (2019)**	pCASL	Pseudo-continuous	3D spiral fast-spin-echo	- TR = 4844 ms<br>- TE = 10.5 ms<br>- 512 sampling points on 8 spirals<br>- Slice thickness = 4.0 mm<br>- NEX = 3	240 x 240 mm	3.64 x 3.64 x 4	n.r.	2000	nr
**Schröder (2019)**	multi-PLD pCASL	Pseudo-continuous	3D-GRASE	- Matrix size = 64 x 48 x 20<br>- Echo train split into 2 segments in-plane<br>- EPI factor = 25<br>- Turbo factor = 20	256 x 192 x 80 mm	4	1500	300, 600, 900, 1200, 1500, 1800, 2100, 2400, 2700, 3000	n.r.
**Soman (2020)**	pCASL	Pseudo-continuous	n.r.	- Slice thickness = 4–5 mm<br>- Spiral trajectory with 6–8 arms<br>- Number of averages = 3–4<br>- Bandwidth = 62.5 kHz	240 x 240 mm	4 or 5	1450	2525	n.r.
**Endo (2020)**	multi-PLD pCASL	Pseudo-continuous	n.r.	- TR = 4546 ms<br>- TE = 10.5 ms<br>- Section thickness = 4 mm<br>- NEX = 2<br>- Bandwidth = 62.50 Hz	240 x 240 mm	4	2.025	1525, 2025, 2525	10 min

**Wang (2021)**	pCASL	Pseudo-continuous	3D background suppressed fast-spin-echo stack-of-spiral	- TR = 4844 ms<br>- TE = 10.5 ms<br>- Slice thickness = 4.0 mm<br>- NEX = 3	240 x 240 mm	3.64 x 3.64 x 4	n.r.	n.r.	n.r.
**Fan (2021)**	pCASL	Pseudo-continuous	3D fast-spin-echo stack-of-spiral	- TR = 4886 ms<br>- TE = 10.5 ms<br>- Slice thickness = 4 mm<br>- In-plane spiral number = 8	240 x 240 mm	3.75 x 3.75 x 4	1450	2025	n.r.
**Fan (2022)**	pCASL	Pseudo-continuous	3D stack-of-spirals FSE	- In-plane spiral arms number: 8<br>- Number of slices: 40<br>- Slice thickness: 4 mm<br>- TR: 4886 ,s<br>- TE: 10.5 ms	240 x 240 mm2	3.75 x 3.75 x 4	1450	2025	n.r.
**Liu (2022)**	pCASL	Pseudo-continuous	n.r.	- TE: 10.5 ms<br>- TR: 4632 ms<br>- Slices thickness: 4 mm<br>- Slice interval: 0	n.r.	2 x 2 x 4	n.r.	2000	3.8 min
**Fan (2023)**	pCASL Hadamard encoded	Pseudo-continuous (modified)	n.r.	- TR = 6023 ms<br>- TE = 10.5 ms<br>- Slice thickness = 4 mm<br>- In-plane spiral arms number = 6	240 x 240 mm	3.75 x 3.75 x 4	n.r.	1000, 1570, 2460	3.6 min
**Xu (2023)**	pCASL	Pseudo-continuous	3D background suppressed spiral fast-spin-echo	- TR: 4632 ms (PLD = 1500 ms) and 4842 ms (PLD = 2000 ms)<br>- TE: 10.5 ms<br>- Labeling Plane: Positioned perpendicular to the carotid arteries around C2/C3	250 x 250 mm	2 x 2 x 4	1500	1500, 2000	- 3.15 min (PLD = 1500 ms)<br>- 3.44 min (PLD = 2000 ms)
**Lindner (2024)**	pCASL	Pseudo-continuous	3D-GRASE backgroud suppressed	- TR = 4000 ms<br>- TE = 12.06 ms	n.r.	3.6 x 3.6 x 4	1500	300, 600, 900, 1200, 1500, 1800, 2100, 2400, 2700, 3000	n.r.

Legend. ASL: arterial spin labeling; CASL: continuous ASL; QUIPPS: quantitative imaging of perfusion using a single subtraction; PASL: pulsed ASL; VE: vessel-encoded; Q2TIPS: second version of quantitative imaging of perfusion using a single subtraction; pCASL: pseudo-continuous ASL; ss: stack of spirals; PLD: pulse-labeling delay; GRASE: gradient and spin-echo; RF: radiofrequency; TR: repetition time; TE: echo time; NEX: number of excitations; FA: flip angle; EPI: echo-planar imaging; FOV: field of view; n.r.: not reported; TI: inversion time; MRI: magnetic resonance imaging; CAS: carotid artery stenting; CEA: carotid endarterectomy; SPECT: single photon emission computed tomography; ACZ: acetazolamide;

Table 3. Findings summary of the included studies.

Author (year of publication)	Features extracted	Analysis tool	ASL Analysis	Study summary
**Ances (2004)**	CBF	IDL	Manual ROIs drawn over CBF on major vascular territories	The lower the pre-surgery CBF in the anterior circulation territory, the higher the increase of CBF after surgery
**Jones (2006)**	- CBF<br>- OR<br>- ORexcess (= ORipsilateral - ORcontralateral)	SPM<br>MATLAB	Manual ROIs drawn over CBF on major vascular territories	The greater the difference in stenosis between the pathological and healthy sides, the more the perfusion via collateral circulation will decrease post-surgery.
**Van Laar (2007)**	- rCBF<br>- extent of perfusion territories	SPM	Manual ROIs drawn over CBF on major vascular territories	Both CAS and CEA lead to an increase and normalization of rCBF, with no significant differences between the two treatments.
**Dang (2012)**	- CBF (from right ICA, left ICA, VAs, and ECA)<br>- extent of perfusion territories (from VE-ASL and DSA)	MATLAB	Manual ROIs drawn over CBF on major vascular territories	After CAS there was an increase in perfusion from the treated ICA and a decrease in collateral flow from the ECA.
**Yun (2013)**	- CBF<br>-rCBF<br>- ΔrCBF	SPM<br>MATLAB	Automatic segmentation of the CBF maps with 90 MNI atlas templates	Patients with greater pre-procedure perfusion deficits experience the most significant post-surgery improvement, more pronounced in eloquent brain regions in the ICA perfusion territory.
**Chen (2016)**	CBF	GE Functool	Manual segmentation of the MCA flow territory on CBF maps on a GE workstation	A significant increase in CBF values was observed after stenting. However in patients with high stenting position, lower post-CAS CBF values due to distortion artifacts in the labeling plane were found.
**Wang (2017)**	- rCBF<br>- Cognition assessment (MoCA Beijing Version, MMSE, DSR, RAVTL, VMT)	SPM	Automatic segmentation of the CBF maps with MNI atlas templates	After CAS increase in CBF in the left frontal gyrus, anterior cingulate, left occipital gyrus, and left cerebellum, without significant correlation with cognitive testing.
**Haga (2019)**	-Arterial Transit Artifacts (ATA),<br>- CBF	SYNAPSE<br>VINCENT<br>E. CAM signature	Visual inspection of CBF maps from two different PLDs	The subtraction of ASL images related to two different PLD can help to distinguish the signal from ATA from post-CEA hyperperfusion (CH)
**Lin (2019)**	- CBF<br>- CoV of CBF<br>- whole brain PV (PV)<br>- Ratio PV (= PV / BV)<br>—<br>- Ipsilateral PV (PVip)<br>- contralateral PV (PVco)<br>- vertebrobasilar PV (PVvba)<br>- Asymmetry index (AIco/ip = PVco/PVip)<br>- Ipsilateral perfusion volume ratio (RatioPVip = PVip/BV)<br>- Perfusion volume ratio apart from ipsilateral side (RatioPVrest = (PVco + PVvba)/BV)	SPM	Automatic segmentation of grey and white matter masks over the CBF maps	Two types of hyperperfusion were observed:<br>- A significant increase in perfusion signal on the intervention side, with absolute CBF higher than the contralateral side.<br>- A clear area of low preoperative perfusion that resolved post-intervention with increased CBF, making the postoperative CBF comparable to the contralateral side.<br>Patients with AcomA or A1 ipo-aplasia exhibited cerebral had higher incidence of hyperperfusion. Patients with hyperperfusion had a significantly higher CBF CoV and a lower RatioPV pre-intervention.
**Lan (2019)**	CBF	GE Functool<br>SPM<br>MATLAB	Automatic segmentation of the CBF maps using a T1w atlas	CBF increased after CAS and CEA both ipsilaterally and contralaterally (although less pronounced), with a peak at 72h after CAS and 48h after CEA
**Schröder (2019)**	- ASL-CBF<br>- ASL-BAT<br>- CE perfusion (CE-CBF, CE-MTT, CE-CBV)<br>- Cognitive testing (MMSE, DemTect, Clock- Drawing Test, Trail-Making Test, Stroop Test	FMRIB BASIL Toolbox	Automatic segmentation of the CBF maps with MNI atlas templates of the vascular territories	Perfusion alterations were observed in the hemisphere ipsilateral to the stenosis, most pronounced in the MCA border zones, and normalized after revascularization. No association between hypoperfusion and cognitive performance were found.
**Soman (2020)**	- CBF<br>- CV risk factors	SPM	Automatic segmentation of the CBF maps with MNI atlas templates	CV risk factors (hypertension, chronic kidney disease, history of stroke) are associated with a smaller increase in CBF after CEA/CAS.
**Endo (2020)**	- CBF (ASL and SPECT)<br>- Asimmetry index (= CBF in the affected MCA territory/CBF in the unaffected MCA territory x 100)<br>- Slope index (slope of the CBF regression at 3 different PLDs)<br>- CVR (SPECT)	FALCON	ROI over the MCA territory on the CBF maps using an automatic method	The slope index calculated from ASL with multiple PLDs is a reliable parameter for predicting CH, yielding results similar to rCBF obtained from ASL with a single PLD or from SPECT with or without an ACZ challenge.
**Wang (2021)**	- CBF<br>- rCBF	GE Functool<br>MATLAB<br>DPABI	Automatic segmentations of frontal, parietal and temporal lobes using an automatic method	Post-stenting (days 1-3), CBF was significantly higher in both ipsilateral and contralateral frontoparietal lobes compared to the temporal lobe, normalizing by day 4. Patients with microembolism** had significantly lower frontoparietal rCBF reduction before surgery
**Fan (2021)**	CBF	GE Functool	Manual segmentation of the CBF maps on a GE workstation	WMH and lacunes were independently associated with postoperative CH. ROC curve analysis showed that a Fazekas score (sum of DWM and PVWM) ≥3 points and the presence of ≥2 lacunes were optimal cutoff values for predicting CH.
**Fan (2022)**	- ATAs<br>- CBF<br>- hyperperfusion index (HI) (CBF post-op /CBF pre-op x voxel volume)	AW Workstation, GE Healthcare<br>MATLAB<br>SPM	Automatic segmentation of vascular territories using an MNI atlas template	Carotid near occlusion, posterior communicating artery opening with incomplete anterior semicircle, and leptomeningeal collaterals were linked to lower ASL ATA scores. The preoperative ASL ATA score independently predicted cerebral hyperperfusion with a cutoff of 25 points (AUC = 0.98, 94.1% sensitivity, 88.4% specificity).
**Liu (2022)**	- rCBF<br>- Plaque burden and the presence of lipid-rich necrotic core, intraplaque hemorrhage, calcifications, ulcer and fibrous-cap rupture<br>- Volume and maximum plaque components' area percentages	GE AW	Manual segmentation of the CBF maps on a GE workstation	Carotid plaque composition, especially IPH, is associated with perioperative reduction in CBF in carotid stenosis patients. Larger IPH may predict less improvement in CBF after CEA.

**Fan (2023)**	- CBF<br>- rCBF<br>- ATT<br>- rATT<br>- ASL ATA visual score	SPM<br>MATLAB	Visual inspections on the perfusion images and ROI over the MCA territory on the CBF map using an automatic method	Higher blood pressure and near-carotid occlusion (NO) were clinical risk factors for postoperative hyperperfusion. An ASL ATA perfusion score <1, CBF <29.98 mL/100 g/min, prolonged ATT >1607.18 ms, lower rCBF, and higher rATT were independent predictors of hyperperfusion.
**Xu (2023)**	- CBF (ASL and CTP)<br>- rCBF (ASL and CTP)<br>- difference ratio of CBF (DRCBF) (ASL and CTP)	SPM<br>MATLAB	Manual segmentation of the MCA territory using superimposition between CBF maps and T1w images	No significant differences were found in pre- and postoperative rCBF measurements between ASL and CTP, suggesting that ASL might be a less invasive alternative to CTP
**Lindner (2024)**	CBF	FMRIB BASIL Toolbox<br>MATLAB	ROIs manually drawn on the entire left and right hemisphere on the CBF maps	The acquisition of 2 PLDs does not influence changes in CBF, suggesting no additional values of dual PLDs over mono PLD in clinical setting.

***CBF increas > 110% after procedure; **development of new DWI lesion after surgery**

Legend. PLD: pulse-labeling delay; CAS: carotid artery stenting; CEA: carotid endarterectomy; SPECT: single photon emission computed tomography; ACZ: acetazolamide; CBF: cerebral blood flow; OR: obstruction ratio; rCBF: relative CBF; ICA: internal carotid artery; VA: vertebral artery; ECA: external carotid artery; DSA: digital subtraction angiography; MoCA: Montreal cognitive assessment; MMSE: mini mental state evaluation; DSR: dementia rating scale; RAVLT: Rey auditory verbal learning test; VMT: vigilance and memory test; CoV: coefficient of variation; PV: perfusion volume; BV: brain volume; ip: ipsilateral; co: contralateral; BAT: bolus arrival time; CE: contrast-enhanced; CV: cardiovascular; MCA: middle cerebral artery; CVR: cerebrovascular reactivity; ATT: arterial transit time; rATT: relative ATT; CTP: computed tomography perfusion; AcomA: anterior communicating artery; ACA: anterior cerebral artery; DWI: diffusion-weighted imaging; WMH: white matter hyperintensities; DWM: deep white matter; PVWM: periventricular white matter; CH: cerebral hyperperfusion.
